# High incidence of occult familial *SDHD* cases amongst Czech patients with head and neck paragangliomas

**DOI:** 10.3389/fendo.2023.1278175

**Published:** 2023-12-08

**Authors:** Anasuya Guha, Ales Vicha, Tomas Zelinka, Martin Kana, Zdenek Musil, Karel Pacak, Jan Betka, Martin Chovanec, Jan Plzak, Jan Boucek

**Affiliations:** ^1^ Department of Otorhinolaryngology, Charles University, 3^rd^ Faculty of Medicine and University Hospital Kralovske Vinohrady, Prague, Czechia; ^2^ Department of Pediatric Hematology and Oncology, Charles University, 2^nd^ Faculty of Medicine and University Hospital Motol, Prague, Czechia; ^3^ 3^rd^ Department of Medicine, Department of Endocrinology and Metabolsim of the 1^st^ Faculty of Medicine and General University Hospital in Prague, Prague, Czechia; ^4^ Department of Otorhinolaryngology and Head and Neck Surgery, Charles University, 1^st^ Faculty of Medicine and University Hospital Motol, Prague, Czechia; ^5^ Institute of Biology and Medical Genetics of the 1^st^ Faculty of Medicine and General University Hospital in Prague, Prague, Czechia; ^6^ Section of Medical Neuroendocrinology, Eunice Kennedy Shriver National Institute of Child Health and Human Development, National Institutes of Health, Bethesda, MD, United States

**Keywords:** HNPGL, CBPGL, paraganglioma syndrome, germline mutation, *SDHD* gene

## Abstract

**Introduction:**

Head and neck paragangliomas (HNPGLs) are rare neuroendocrine tumors, which are mostly benign in nature. Amongst all genes, Succinate Dehydrogenase Subunit D (*SDHD*) is the most commonly mutated in familial HNPGLs. In about 30% of HNPGLs, germline mutations in *SDHD* can also occur in the absence of positive family history, thus giving rise to “occult familial” cases. Our aim was to evaluate the pattern of *SDHD* germline mutations in Czech patients with HNPGLs.

**Materials and methods:**

We analyzed a total of 105 patients with HNPGLs from the Otorhinolaryngology departments of 2 tertiary centers between 2006 – 2021. All underwent complex diagnostic work-up and were also consented for genetic analysis.

**Results:**

Eighty patients aged 13-76 years were included; around 60% with multiple PGLs were males. Carotid body tumor was the most frequently diagnosed tumor. Germline *SDHD* mutation was found in only 12% of the Czech patients; approximately 78% of those harboring the mutation had negative family history. The mutation traits had higher affiliation for multiple tumors with nearly 70% patients of ≤ 40 years of age.

**Conclusion:**

An *SDHD* mutation variant was shared amongst unrelated patients but no founder-effect was established. Our findings confirmed that the pattern of *SDHD* mutation distribution amongst HNPGLs in Czech Republic differs from most studies worldwide.

## 1 Introduction

Head and neck paragangliomas (HNPGLs) are classified as tumors originating from extra-adrenal paraganglias (1, 2), with an overall incidence of 0.3 to 1 in 100 000 (3, 4). Gender distribution shows a higher female predominance of 3-4:1; most patients become symptomatic between their fourth and seventh decade of life (5, 6). Carotid body paragangliomas (CBPGLs) represent 60% of all types of HNPGLs, with bilateral presentation in about 10% of patients (5, 7, 8). Other frequently detected HNPGLs include jugulotympanic (<35-40%) (9) and vagal (<5%) (10). These neuroendocrine tumors are mostly benign and non-secretory in nature. Patients can remain asymptomatic for long periods, however, the tumors can be found incidentally either during ultrasound of the neck or due to symptoms arising from cranial nerve dysfunction (6). A multidisciplinary approach is required for the management of such tumors.

This disease can occur in a sporadic or hereditary form, hence genetic analysis plays a pivotal role in differentiating the forms. This form of investigation has been popularized for the early detection, management and prediction of tumors in familial cases. It is also now known that nearly 40-50% of all HNPGLs are hereditary (11), including a significant subset without known family history.

Although many genes have been linked to pheochromocytomas and paragangliomas, the mitochondrial complex II genes, subunits of Succinate Dehydrogenase (*SDHx*) genes, have been most often identified as susceptible to the development of HNPGLs (5, 6). The discovery of Succinate Dehydrogenase Subunit D (*SDHD*) gene in families with Paraganglioma syndrome type 1 (PGL1) in 2000, helped in understanding the molecular mechanism of paraganglioma inheritance (4, 6, 12). Subsequently, it was shown, that mutations in other subunits (A – C) of *SDH_x_* along with the *SDH* Assembly Factor (*SDHAF2*) genes lead to Paraganglioma syndrome types 2 to 5 (PGL2-5), which are all inherited in an autosomal dominant manner (3–7). In hereditary syndromes, jugular, vagal and carotid PGLs are observed in 26%, 31%, and 39% of cases respectively (3, 13). Young age (≤ 40 years) with multiple tumors, positive family history, presence of carotid body tumor as well as bilateral presentation have a higher predilection for familial forms of the disease (6, 9, 13, 14). PGL1, related to the *SDHD* gene, has the highest affinity for HNPGLs (13). Germline mutations in *SDHx* genes occurring in suspected sporadic HNPGLs, due to the absence of positive family history, suggests the possibility of “occult familial” cases. This concept is mainly seen with *SDHD* (14, 15). Similarly, the risk of occult paragangliomas amongst *SDHD* carriers is also relatively high (16). Therefore, the evidence supports the fact, that, patients with head and neck paragangliomas should undergo genetic testing (4, 6, 11, 17).

We decided to study the distribution pattern of *SDHD* mutations amongst our cohort of patients with HNPGLs. From a clinical point of view, these findings will also have an impact on the early management including screening of family members of such patients in standard clinical practice.

## 2 Materials and methods

### 2.1 Patients

Between 2006 and 2021, 105 patients with HNPGLs were referred for consultation to the departments of Otorhinolaryngology across 2 tertiary centers. A multidisciplinary approach was adopted in all patients. Patients underwent standard examination including clinical, biochemical and radiological (anatomical and functional imaging) investigations. The HNPGLs were classified by focality and localization [carotid body (CBPGLs), jugular (JPGLs), tympanic (TPGLs) and vagal (VPGLs)] based on clinical and radiological findings. Furthermore, other forms of paragangliomas and sites of metastases were also identified. Plasma metanephrine, normetanepherine and chromogranin A were used to assess secretory activity of tumors and risk of malignity. The importance and possibility of genetic analysis were discussed with all the patients and referred accordingly. A treatment plan was advocated in each case, with the decision on interventional therapy or ‘wait and scan’ approach.

### 2.2 Protocol for Genetic investigations

Genetic examination was recommended for all our patients and consent was obtained accordingly. Those who did not consent or failed to attend their tests were excluded from this study. Peripheral blood samples were collected to initiate the process. Genomic DNA was extracted from 10mL of EDTA-anticoagulated blood using standardized methods. In our genetic center, we use Sanger Sequencing for single gene mutation analysis to exclude *SDHD* first followed by Next Generation Sequencing (NGS). However, in cases with multiple HNPGLs or if requested by the referring physician, NGS was done first. In the context of sharing similar research interests for pheochromocytomas and paragangliomas (PPGLs), we also performed Whole Exome Sequencing (WES) for 13 patients to compare results with NGS examination in Czech Republic. This was done in collaboration with the National Institutes of Health and National Cancer Institute, Bethesda, USA.

On identification of an index patient with positive germline mutation, they were advised to contact their first degree relatives at risk to undergo genetic counselling and if necessary preventive scanning in order to evaluate carrier status.

#### 2.2.1 Sanger sequencing

The extracted DNA from peripheral blood samples were checked for quality control. These were analyzed using specific primers for SDHD exons 1-4 (primer sequence available on request). DNA fragments were sequenced in both forward and backward directions using an automatic fluorescent ABI Prism™ 3130 Genetic Analyzer (PE Applied Biosystems). DNA sequence analysis was then done using the Mutation Surveyor (Carolina Biosystems^®^, USA) software.

#### 2.2.2 Next generation sequencing

Capture-based next-generation DNA sequencing was performed on a NextSeq 500 instrument (Illumina^®^, San Diego, California, USA). A custom Pheochromocytoma/Paraganglioma panel was used. This covers the entire coding as well as selected intronic and promoter regions of 123 genes, which are of particular relevance in these tumors. Agilent capture system was used (SSEL XT HS Reagent Kit, Agilent). Reads were aligned against the reference genome (GRch38). GENOVESA (BIOXSYS^®^, Czech Republic) software was used for analysis.

#### 2.2.3 Whole exome sequencing

This technique of sequencing consisted two main processes, namely target-enrichment and sequencing. Sample preparation included purification and quality control of DNA samples. The next step was target-enrichment (DNA fragmentation and exome capture). This was performed to select and capture exome from DNA samples. Seventy Exome samples were pooled and sequenced on NovaSeq 6000 S2 (Illumina^®^, USA) run using Agilent^®^ SureSelect Human All Exon V7 and paired-end sequencing mode. The samples have 100M to 189M pass filter reads, with Q30 above 89%. The samples were mapped and variants were called using Dynamic Read Analysis for GENomics (Dragen; Illumina^®^, USA).

## 3 Results

### 3.1 Patient demographics and characteristics of tumors

A total of 105 patients were referred with HNPGLs; however 80 (25 males; 55 females) patients of 13-76 years completed genetic testing. Approximately 40% of patients were ≤ 40 years of age. Seventy-six patients were of Czech origin, the other four were from Poland, Hungary, Slovakia and Syria. Only 7 patients had a positive family history of HNPGLs; six were females. A total of 102 head and neck tumors were found amongst 80 patients; 94% had benign tumors (**Table 1**). Four out of five patients with metastatic disease, had solitary tumors. CBPGLs were the most commonly diagnosed tumors, followed by JPGLs, TPGLs and VPGLs. All patients with positive family history had CBPGLs. Bilateral carotid body tumors were seen in 8 patients; approximately 63% being males. Tympanic paragangliomas were almost exclusively found in females. Amongst 15 patients with multiple HNPGLs, 93% were of Czech origin. Representative data from previously published results on patients with multiple tumors have been included here (18). About 67% of patients with multiple tumors were of young age (≤ 40 years) and had higher male predominance. Five patients had paragangliomas located below the neck; three patients had mediastinal PGLs whilst retroperitoneal tumors were detected in 2 others. Pheochromocytomas were not seen in our cohort. Raised plasma metanephrine and normetanephrine levels were detected in 2 related patients, diagnosed with solitary CBPGL and mediastinal PGL. Amongst those with multiple HNPGLs, Chromogranin A was elevated in two patients (one had retroperitoneal and the other had mediastinal PGL) and Normetanephrine was higher in another patient (18). Amidst those with benign tumors, 64 patients had intervention (89% surgery; 5% radiotherapy; 6% combination therapy), 10 were allocated to ‘wait and scan’ and one patient died from respiratory complications of advanced disease. Those with metastases had combination therapy.

**Table 1:** Characteristics of patients with HNPGLs.

Characteristics		Patients N=80 (%)	Familial cases N=7 (%)	Sporadic cases N=73 (%)
Demographic profile				
Gender	*Males*	25 (31)	1 (14)	24 (33)
	*Females*	55 (69)	6 (86)	49 (67)
Origin	*Czech*	76 (95)	6 (86)	70 (96)
	*Other*	4 (5)	1 (14)	3 (4)
Age of ≤ 40 years		32 (40)	3 (43)	29 (40)
Genetic mutation				
	*SDHD*	11 (14)	3 (43)	8 (11)
	*Other SDHx*	11 (14)	2 (29)	9 (12)
Tumor features				
Focality	*Single*	65 (81)	3 (43)	62 (85)
	*Multiple*	15 (19)	4 (57)	11 (15)
Type	*Benign*	75 (94)	6 (86)	69 (95)
	*Metastatic*	5 (6)	1 (14)	4 (5)
Total head and neck tumors		102	11	91
Localization	*CBPGL*	58 (57)	8 (73)	50 (55)
	*JPGL*	19 (19)	1 (9)	18 (20)
	*TPGL*	15 (14)	0 (0)	15 (16)
	*VPGL*	10 (10)	2 (18)	8 (9)

### 3.2 Germline mutation analysis in HNPGLs

On completion of all genetic analysis, which included the NGS panel genes for PPGLs and in certain cases, the use of the extended genetic library for WES, interestlingly, only *SDH_x_* pathogenic germline mutations were found in the entire cohort. Germline mutations were detected in 22 patients; eight were found with *SDHB* gene mutations, whilst 3 had *SDHC* and only 11 had *SDHD* mutation (**Table 2**). We only reported pathogenic and likely pathogenic variants according to the American College of Medical Genetics and Genomics (ACMG) classification and Clinical Variants Database of germline mutation (ClinVar) database. No novel mutation was found. Nine patients were of Czech origin, one was from Poland and the other was of Slovakian origin. Three patients had positive family history including a patient from Poland (18). The variants of *SDHD* mutation showed a higher affiliation for patients ≤ 40 years old with multiple tumors. Approximately 64% with *SDHD* mutation were females. For the purposes of our study and to accurately assess the frequency of *SDHD* mutation amongst Czech patients, we excluded the 4 patients with other nationalities.

**Table 2:** *SDHD* germline mutation analysis in patients with HNPGLs.

No.	Age (yrs.)	Gender	*SDHD* Mutation Variant	Pathogenecity (ClinVar, ACMG)	Localization of HNPGL(s)
1	23	F	c.361C>T, p.Gln121X	Pathogenic	CBPGL (B), VPGL (L)
2	26	M	c.2T>A, p.Met1Lys	Pathogenic	CBPGL (B), JPGL (B), VPGL (R)
3	29	F	c.341A>G, p.Tyr114Cys	Pathogenic	JPGL (R)
4	35	F	c.361C>T, p.Gln121X	Pathogenic	CBPGL (R), JPGL (L)
5	35	F	c.341A>G, p.Tyr114Cys	Pathogenic	CBPGL (B)
6	36	F	c.361C>T, p.Gln121X	Pathogenic	CBPGL (L)
7	36	M	c.1A>G, p.Met1Val	Pathogenic	CBPGL (B), VPGL (B), JPGL (R)
8	37	M	c.209G>T, p.Arg70Ser	Pathogenic	CBPGL (R)
9	40	M	c.305A>T, p.His102Leu	Pathogenic	CBPGL (B)
10	43	F	c.112C>T, p.R38Ter	Pathogenic	CBPGL (R), VPGL (L)
11	57	F	c.53-2A>G	Likely Pathogenic	VPGL (R), JPGL (R)
		Czech patient with +ve F/H			
		Czech patient with -ve F/H			

*F/H, Family History; B, Bilateral; L, Left; R, Right.

The *SDHD* mutation was found only in 12% of Czech patients, where 78% were occult familial cases (**Table 2**). CBPGLs were seen in 8 out of 9 patients, bilateral tumors being present in 56% cases. The c.361C>T, p.Gln121X variant was reported in 2 familial as well as in a suspected sporadic case. These patients were unrelated. All of them were females below 40 years of age and had CBPGLs. The youngest patient with a positive family history and multiple HNPGLs was diagnosed with metastatic disease. Lymph node, bone and liver metastases were detected on 68Ga-DOTA-TOC PET-CT whole body imaging. The 35-year old female patient with multiple benign HNPGLs also has a sister with multiple paragangliomas. The last patient with the same variant had a single tumor and negative family history. Both children of this 36-year old female patient also tested positive for this mutation, however, they are clinically silent due to the probability of maternal imprinting.

The variant c.341A>G, p.Tyr114Cys causing occult PGL1 in patient no. 5 with benign bilateral CBPGLs was also found in the Slovakian patient with single JPGL. Highest number of tumors including a mediastinal PGL was diagnosed in the 36-year old male patient, who developed very advanced disease that led to his death from severe lower cranial nerve dysfunction (18). The other 4 *SDHD* mutation variants were also occult familial cases with benign tumors. The last patient in this series was diagnosed with the second tumor after 3 years follow-up (18). As already mentioned, eleven patients had *SDHx* gene mutations other than *SDHD*. In comparison to total *SDHx* gene mutations, *SDHD* gene mutation was seen in 47%.

## 4 Discussion

During the period of our study, 80 out of 105 patients diagnosed with HNPGLs were included. Amongst these patients, 95% were of Czech origin. We demonstrated higher female predominance (F:M = 2.2:1), typically seen in HNPGLs, but less than expected for females (5). Fifteen patients had multiple tumors including 5 patients with PGLs below the neck. Only 3 (one with *SDHB* and two family members with *SDHC* germline mutation) patients had elevated catecholamines; Chromogranin A levels were raised in 2 unrelated patients with *SDHD* germline mutation. Approximately 6% of all patients had metastatic disease.

Carotid body tumors (57%) were the most commonly found HNPGLs, followed by JPGLs (19%), TPGLs (14%) and VPGLs (10%). This pattern is in accordance with most reported studies (5, 7, 9, 17). Eight out of fifty patients with CBPGLs, had bilateral presentation. All 7 patients with positive family history including 1 of Polish origin had carotid body tumors (43% solitary; 57% bilateral). These tumors are usually of non-hereditary form in about 60% of the cases (3, 9), a finding confirmed by our study too. It should be mentioned that up to 72% patients diagnosed with *SDHx* germline mutation including nine out of the eleven with *SDHD* gene mutation had an affinity for CBPGLs. Therefore, the presence of carotid body tumor, whether as solitary or bilateral should also be considered a risk for germline disease (19, 20). Jugulotympanic tumors were almost exclusively seen in female patients, an observation made in a large muticentric study too (17). Bilateral jugular PGLs were seen in 2 males only.

Researchers worldwide reported that head and neck paragangliomas (solitary or multiple) represent a strong predictor for *SDHD* mutation even in small cohort of patients (15, 21–32) (**Table 3**). The *SDHD* mutation was surprisingly found in only 9 out of 76 Czech patients.

**Table 3:** Worldwide distribution of *SDHD* germline mutations in patients with HNPGLs.

Authors	Country	Timeline	Patients	% *SDHD*	% *SDHD* with + F/H	% *SDHD* /total *SDHx*
Astuti et al. (21)	United Kingdom	1990-1999	34	12	100	100
Badenhop et al. (22)	Australia	1991-2001	34	32	82	79
Baysal et al. (23)	USA	2002	55	16	50	60
Benn et al. (24)	Australia, Canada, France, Germany, New Zealand, United Kingdom, United States	2003-2004	27	89	79	89
Fakhry et al. (25)	France	1994-2007	23	26	100	75
Guha et al.	Czech Republic	2006-2021	76	12	33	47
Hensen et al. (26)	Netherlands	1950-2009	236	83	93	91
Lima et al. (15)	Spain	1981-2005	40	18	79	35
Neumann et al. (27)	Germany, Poland	2000-2004	83	33	N/A	73
Pandit et al. (28)	India	N/A	10	10	0	100
Papaspyrou et al. (29)	Germany	1989-2010	86	26	100	65
Persu at al (30).	Belgium	2003-2006	36	28	100	53
Piccini et al. (31)	Italy	2003-2011	79	37	100	85
Snezhkina et al. (32)	Russia	N/A	102	25	N/A	61

*F/H, Family History; N/A, Not Available.

It is already well established, that those with the familial form of the disease, usually present at a younger age (less than 40 years) and with multiple tumors (9, 11, 17, 19, 20). Similarly, the *SDHD* variants discovered in our study exhibited a strong association with young age and multiple tumors.

Furthermore, a large number of studies showed that the percentage of germline *SDHD* mutations in positive family history could be as high as 80% to 100% (**Table 3**). This occurrence is most typical for Netherlands and is supported by consistent findings (14, 26, 33). In contrast, only one-third of Czech patients with known family history showed germline pathogenic *SDHD* mutation. Interestingly ‘occult familial cases’ were observed in almost 78% of patients diagnosed with PGL1. This may be partially explained by the pattern of transmission. Theoretically, this autosomal dominant syndrome can be inherited both via the paternal and maternal lines, but in maternal transmission, PGLs almost never develop. There is still a 50% chance of maternally derived carriers transmitting the mutation to their offspring, hence PGL1 can seem to skip generations (3).

The most frequently reported variant amongst Czech patients was the *SDHD* c.361C>T, p.Gln121X, a pathogenic point mutation. This was seen in 2 unrelated familial as well as in a suspected sporadic case; all of them were below the age of 40 years (**Table 2**). This mutation was also observed in another unrelated young male patient from Czech Republic with negative family history and retroperitoneal PGL (34). The youngest patient amongst the three with c.361C>T, p.Gln121X in this study, had a positive family history presented with multiple tumors and metastatic liver disease, which is an unusual feature. This sort of uncommon presentation was also seen in a family in Brazil; all diagnosed members were of young age. Apart from HNPGLs, pheochromocytomas were also seen. Here, the youngest of three members, an 11-year old boy was also diagnosed with metastatic PPGL affecting the lung (35). Despite our findings, a founder effect in Czech Republic related to c.361C>T, p.Gln121X could not be established.

The variants diagnosed in patients no. 7, 10 and 11 have been discussed in detail in a previous study (18). The 36-year old male patient with c.1A>G, p.Met1Val, initially diagnosed with jugular tumor, had rapid progression over a span of 10 years leading to the development of 5 more PGLs. No signs of metastasis was detected. The oldest patient with c.53-2A>G also primarily presented with a solitary VPGL.

The c.341A>G, p.Tyr114Cys protein variant was found in 2 unrelated young female patients of different origins, but of close geographical locations. The Czech patient had bilateral CBPGLs, whilst the Slovakian patient had solitary JPGL. This is a missense mutation of pathogenic variant. This was reported in a large study from Italy, showing the endemic nature of PGL1 in Trentino natives, thus accounting for one of the oldest and largest *SDHD* founder effect ever seen (36). The other missense mutation c.209G>T, p.Arg70Ser, a variant of the p.R70M, was related to patient no. 8 with solitary CBPGL. This has also been reported in several studies (19, 30, 37, 38). On a retrospective study done from the Mayo Clinic, the c.305A>T, p.His102.Leu was identified in a 30-year old with a single CBPGL (39), findings being similar to our 40-year old patient with bilateral carotid body tumors.

Metastasis with *SDHD* mutation was detected in 1 Czech patient with positive family history. This is rarely seen in patients with HNPGLs, and is an even more unexpected finding in relation to PGL1. As such predisposition to malignancy with hereditary background is highest amongst those with *SDHB* mutation, and about 1-3% with *SDHD* (18, 40), which is synchronous with our findings.

Lastly, comparative analysis indicated that most studies have a high rate of *SDHD* mutations in comparison to other *SDHx* gene mutations in the pathogenesis of HNPGLs; at least 8 out of 13 studies showed a ratio of above 70% (21, 22, 24–28, 31) (**Table 3**). This proportion was less than 50% amongst our patients, as seen in the Spanish cohort (15).

We could contemplate that patients may be unaware of their family history or their family members remained asymptomatic and therefore undiagnosed. The disease being mostly of benign nature and slow progression, most patients may find it difficult to understand the risk associated with transmitting the mutation. The one major limitation of our study was the inability to test most of the at-risk first-degree relatives, despite index patients receiving genetic counselling. It has been proposed that the probability of ascertaining a mutation decreases to 40% in patients without a family history (33). Here, another factor to consider would be the migration trend of Czech inhabitants to other countries, which might have an impact on research associated with such disease.

The importance of the genetic mutation profile we carried out amongst our patients not only demonstrated a low absolute frequency of *SDHD* gene mutation amongst the Czech population, but also showed inconsistency in patients with known family history. In comparison to most studies, there is a significant discrepancy that arose between the expected and actual outcome in terms of observed frequency of *SDHD* mutation. The ratio of *SDHD* gene in comparison to total *SDHx* gene mutations was also lower. More importantly, we also determined a high incidence of ‘occult familial cases’, which is not a common phenomenon for HNPGLs. It should be considered, that there is a high prevalence of occult paragangliomas in asymptomatic carriers of *SDHD* and *SDHB* gene mutations. As such, one clinical surveillance disclosed that up to 59.6% of asymptomatic *SDHD* carriers can have occult HNPGLs (16). Absence of family history does not rule out the presence of germline mutations in *SDHx* genes, especially *SDHD*. Patients with undetected germline mutations are not only at risk of developing multiple tumors but may also transmit the mutation to the next generation (33, 41). Despite a number of recommendations being suggested regarding early detection of HNPGLs and determination of genetic profile, the uncommonness of these tumors and delayed presentation will always present a certain risk of underestimated cases reported in any cohort.

## 5 Conclusion

Germline *SDHD* gene mutation was found in only 12% of all Czech patients and 78% could be described as ‘occult familial cases’. We were able to establish the relationship between germline *SDHD* and the presence of multiple tumors in younger patients, but with known family history, the affinity was lower. Our results showed a different pattern in comparison to other studies worldwide. The *SDHD* c.361C>T, p.Gln121X variant was the most frequently detected mutation in Czech patients, however a founder effect was not established. Therefore, the key to prediction and early management of HNPGLs should include reiterating the importance of genetic testing to patients and ascertaining a comprehensive guidance protocol for all physicians involved in the care of such patients.

## Data availability statement

All relevant data is contained within the article: The original contributions presented in the study are included in the article/supplementary material, further inquiries can be directed to the corresponding author.

## Ethics statement

All procedures performed in studies involving human subjects were in compliance with the Helsinki declaration and further in accordance with local ethical guidelines of the institutional ethical committees of Charles University, Prague, Czech Republic. Informed consent was obtained for all patients undergoing intervention according to the individual hospital regulations, institutional guidelines of Charles University and those defined by the practice codes of the Ministry of Health of the Czech Republic. Additional informed consent was obtained for genetic testing of patients. No identifying information of the patients have been included in this manuscript.

## Author contributions

AG: Conceptualization, Data curation, Formal analysis, Methodology, Project administration, Resources, Supervision, Validation, Writing – original draft. AV: Data curation, Funding acquisition, Investigation, Methodology, Resources, Software, Validation, Writing – review & editing. TZ: Funding acquisition, Investigation, Methodology, Resources, Validation, Writing – review & editing. MK: Data curation, Methodology, Resources, Writing – review & editing. ZM: Investigation, Resources, Software, Validation, Writing – review & editing. KP: Formal Analysis, Investigation, Methodology, Resources, Writing – review & editing. JBe: Funding acquisition, Methodology, Resources, Writing – review & editing. MC: Data curation, Investigation, Methodology, Resources, Writing – review & editing. JP: Data curation, Funding acquisition, Methodology, Resources, Writing – review & editing. JBo: Data curation, Funding acquisition, Methodology, Supervision, Validation, Writing – review & editing.
